# Factors Associated with the Timing of Initial Visit to Healthcare Providers for Injured Workers with Low Back Pain Claims: A Multijurisdiction Retrospective Cohort

**DOI:** 10.1007/s10926-025-10268-5

**Published:** 2025-01-13

**Authors:** Tesfaye Hambisa Mekonnen, Grant Russell, Luke R. Sheehan, Alex Collie, Michael Di Donato

**Affiliations:** 1 Department of General Practice, School of Public Health and Preventive Medicine, Monash University, 553 St Kilda Road, Melbourne, VIC 3004 Australia; 2 Department of Environmental and Occupational Health and Safety, Institute of Public Health, College of Medicine and Health Sciences, University of Gondar, P.O. Box. 196, Gondar, Ethiopia; 3 Healthy Working Lives Research Group, School of Public Health and Preventive Medicine, Monash University, 553 St Kilda Road, Melbourne, VIC 3004 Australia

**Keywords:** Health services accessibility, Low back pain, Occupational injuries, Primary health care, Time to treatment, Workers’ compensation

## Abstract

**Purpose:**

Evidence shows that patient outcomes following musculoskeletal injury have been associated with the timing of care. Despite the increasing number of injured workers presenting with low back pain (LBP) in primary care, little is known about the factors that are associated with the timing of initial healthcare provider visits. This study investigated factors that are associated with the timing of initial workers’ compensation (WC)-funded care provider visits for LBP claims.

**Methods:**

We used a retrospective cohort design. A standardised multi-jurisdiction database of LBP claims with injury dates from July 2011 to June 2015 was analysed. Determinants of the time to initial general practitioner (GPs) and or musculoskeletal (MSK) therapists were investigated using an accelerated failure time model, with a time ratio (TR) > 1 indicating a longer time to initial healthcare provider visit.

**Results:**

9088 LBP claims were included. The median time to first healthcare provider visit was 3 days (interquartile range (IQR) 1–9). Compared to General practitioners (GPs) (median 3 days, IQR 1–8), the timing of initial consultation was longer if the first healthcare providers were MSK therapists (median 5 days, IQR 2–14) (*p* < 0.001). Female workers had a shorter time to first healthcare provider visit [TR = 0.87; 95% CI (0.78, 0.97)] compared to males. It took twice as long to see MSK therapists first as it did to see GPs for injured workers [TR = 2.12; 95% CI (1.88, 2.40)]. Professional workers and those from remote areas also experienced delayed initial healthcare provider visits.

**Conclusions:**

The time to initial healthcare provider visit for compensable LBP varied significantly by certain occupational and contextual factors. Further research is needed to investigate the impact of the timing of initial visits to healthcare providers on claim outcomes.

**Supplementary Information:**

The online version contains supplementary material available at 10.1007/s10926-025-10268-5.

## Introduction

Low back pain (LBP) is a widespread musculoskeletal condition that substantially contributes to the global disability burden [1–3]. LBP presents remarkable personal and societal burdens in terms of severity, quality of life, function and work capacity, and healthcare costs and productivity losses [4]. Back pain was the major reason for years lived with disability, accounting for 7.4% of years lived with disabilities in 2022 worldwide [2, 5]. In Australia, back problems were responsible for the third most prevalent non-fatal disease burden in 2023 [6]. The treatment and management of LBP accounted for 2.2% of Australia’s total healthcare costs in 2020–21 [6]. LBP is a common reason for workers’ compensation claims [7]. Early appropriate interventions for work-related injuries, including LBP, can reduce disability and associated costs [8, 9].

Several nations fund income benefits, healthcare and rehabilitation services to injured workers who sustain work-related injuries and conditions to accelerate their recovery and return to work via workers’ compensation (WC) schemes [10, 11]. Eleven major WC schemes operate independently in Australia, one scheme for each of the eight states and territories and three schemes national for government and large organisations [12]. In Australia, general practitioners (GPs) are often the initial point of contact for medical care for injured workers navigating the WC system. However, workers are generally free to choose other primary care clinicians, such as MSK therapists, as their initial healthcare provider without first consulting a GP, for the management of their injuries and conditions [13]. GPs play a significant role in determining injured worker capacity (i.e. issuing initial and ongoing certificates of capacity) [14]. In some WC schemes (e.g. the state of Victoria), MSK therapists (e.g. physiotherapists) may be able to assess an ongoing capacity for injured workers. MSK therapy services, including physiotherapy, osteopathy, and chiropractic care, are also widely used for managing injured workers presenting with LBP in Australia [15].

Timely care of injured workers, from the initial report of injury or illness to the first healthcare consultation, has been shown to improve patient outcomes [16, 17]. For example, evidence-based physical therapy and biopsychosocial interventions delivered soon after a LBP onset have been associated with a more rapid return to work [16, 18]. While timely, evidence-based care is generally recognised for improved outcomes, certain administrative requirements [19, 20] and varying levels of understanding of compensation processes among healthcare providers [21] may introduce delays in timely workers’ compensation-funded care. Conversely, delayed access to appropriate care is associated with adverse outcomes and increased costs [22]. Studies in North America show that a delay in the first post-injury healthcare consultation was associated with a longer disability duration for LBP claims [23, 24]. A systematic review study by Burns et al. demonstrates that a delay between injury onset and the first medical care is associated with reduced overall quality of life [25]. This emphasises the importance of timely healthcare access for recovery, return to work and overall individual well-being. However, it is also important to note that excessive or overly early treatment for certain musculoskeletal conditions may be associated with adverse patient outcomes [26].

A range of factors have been proposed to significantly influence the timing of care in WC schemes. A previous scoping review identified that certain individual and contextual factors are significantly associated with the timing of healthcare consultation in compensable musculoskeletal conditions [16]. The review included studies primarily from North America or those that did not consider specific healthcare services or conditions. The current study aimed to determine, in a population of workers reporting work-related LBP, the factors associated with the median time to initial visit to GPs and MSK therapists. Given that LBP is costly and disabling, understanding when individuals seek care, and the factors influencing this decision is imperative to improve access to care and identify potential barriers or facilitators of care timeliness within WC systems.

## Methods

### Study Design

This study employed a retrospective cohort design using a standardised multi-jurisdictional WC database (MJD) containing accepted LBP claims from different states in Australia, including Victoria, South Australia, and Western Australia [27]. Patient-reported dates of onset range from July 2011 to June 2015.

### Setting

This study was conducted within the WC settings using the administrative MJD [27]. The administrative data include claim-level and service-level datasets from states and central government WC regulators in Australia. Claim-level data include variables such as a unique claim identifier, data provider, sex, age, occupation, socioeconomic status, remoteness, and time loss in weeks. Service-level data contain information such as jurisdiction, service date, injury date, cost, and service descriptions (e.g. type of healthcare providers). The detailed data cleaning and standardisation procedure has been reported previously [14, 27, 28].

### Sample Selection

This study examined WC claims from two Australian jurisdictions (Western Australia and South Australia) with a patient-reported date of onset ranging from 01 July 2011 to 30 June 2015. Total workers in these two jurisdictions account for 18% of the Australian labour force [29]. Claims from other jurisdictions in the multi-jurisdictional database were excluded due to data availability and, in the case of Victoria, because of a state-specific medical excess policy that may have affected the findings. In contrast to other jurisdictions, Victoria mandates that employers must pay a certain amount of medical expenses (adjusted annually) before insurance benefits can be accessed. This information is not included in the MJD.

We included accepted WC claims for LBP involving workers aged 15–80 years with at least ten consecutive days off work following the reported date of LBP onset. The Type of Occurrence Classification System (TOOCS) [30], which has been used in previous works was used to define cases of LBP [10, 28]. Included services were those that occurred within 730 days (2 years) following the patient-reported onset of LBP. A two-year follow-up window period was used because wage replacement benefits end after two years in some workers’ compensation schemes.

Only services involving patient interactions with clinicians were considered. Patient interaction involves direct contact between the healthcare provider and the worker (e.g. in clinics, via telehealth) [12, 14, 27, 31]. This typically includes services provided by GPs, physiotherapy, osteopathy, and chiropractors. Chiropractic and osteopathy services together accounted for 4.5% of the total service volume, and the guideline adherent services provided by these professions would be broadly similar to those offered by physiotherapy. We also found no significant differences in the timing of initial healthcare provider visits between those groups (Kruskal–wallis; ch^2^ = 0.043, *p* = 0.997). Therefore, we grouped physiotherapists, chiropractors and osteopaths as MSK therapists.

### Study Variables

#### Outcome

*Time to initial healthcare provider visit* Our principal outcome of interest was time to initial healthcare provider visit, defined as the time interval (in days) between the patient-reported onset of LBP and the first consultation with GPs or MSK therapy funded by a WC scheme.

### Independent Variables

Potential covariates were selected based on their availability within the MJD and similar prior studies [32]. Sex is dichotomised as males versus females. Age is collapsed into five categories: 15–24 years, 25–34 years, 35–44 years, 45–55 years and 55+ years, derived from the workers’ age at the time of injury, following previous publications [31, 32]. Workers’ socioeconomic status is described as the most disadvantaged for scores within the lowest quintiles and as most advantaged for scores within the highest quintiles, based on the Index of Relative Socioeconomic Advantage and Disadvantage [33]. Following a previous study [32], the middle quintiles are combined, forming a single category. We measured remoteness using the Accessibility or Remoteness Index of Australia (ARIA) which is grouped into major cities, regional, and remote [34]. Occupational groups are classified using the eight main occupational codes developed by the Australian and New Zealand Standard Classification of Occupations (ANZSCO) [35]. The year of injury was extracted from the claim-level data using the patient-reported injury onset variable. The type of the first healthcare provider was extracted from the service-level dataset. Finally, the WC jurisdictions within which the claims were reported include South Australia and Western Australia [32].

### Data Analysis

A small portion of claims with missing socioeconomic and remoteness data (< 5%) were imputed using multiple imputations (with 10 iterations) by the chained equations method [36]. A careful examination of the missing patterns showed that data were missing at random [37].

We used descriptive analysis to summarise the data. Frequencies and percentages were used to describe categorical variables, while medians with interquartile ranges (IQR) were employed to summarise continuous data. Since the time to first care exhibited a non-normal distribution, the medians (in days) were used as the summary measure. Sample characteristics were reported using frequency tables, and histograms were employed to visualise the distribution of median time to the first visit by healthcare provider type.

As the assumption of proportional hazards was violated in our analysis, an accelerated failure time (AFT) model was employed to identify the factors associated with time to first visit to a healthcare provider [37, 38]. The AFT models do not require proportional hazard assumptions [39]. We employed two most commonly used AFT models, lognormal and Weibull, based on the distribution of the data (lower Akaike Information Criterion (AIC) and Bayesian Information Criteria (BIC) values indicate a better-fit model) [37, 38].

Three different models were fitted: one for the overall time to first visit and one each for GPs and MSK therapy subsamples. A service-specific model was designed because GPs and MSK therapists represent the most common service providers for patients with compensable LBP and warrant investigation. Lognormal distributions were used for the first two models, while the subsample of claimants who saw MSK therapists first employed the Weibull distribution.

In all models, days to the first visit to healthcare providers were censored at 30 days following the date of injury. We chose a 30-day time frame because we anticipate that most initial consultations with care providers occur within a few weeks of the reported date of LBP onset, consistent with previous studies [18, 24]. Covariates were entered into the models simultaneously without using pre-selection criteria. The reference category for each covariate was chosen based on the largest sample size within that variable. Time ratios (TR) and their corresponding confidence interval (CI) of 95% were reported with a TR > 1, indicating a longer time to the first visit. Adjusted survival curves comparing the timing of first visit by care provider and MSK therapy timing by jurisdiction were plotted. A statistically significant level was defined at a *p* < 0.05 value. Stata version 17 was used for analyses.

## Results

### Sample Characteristics

**Fig. 1:**
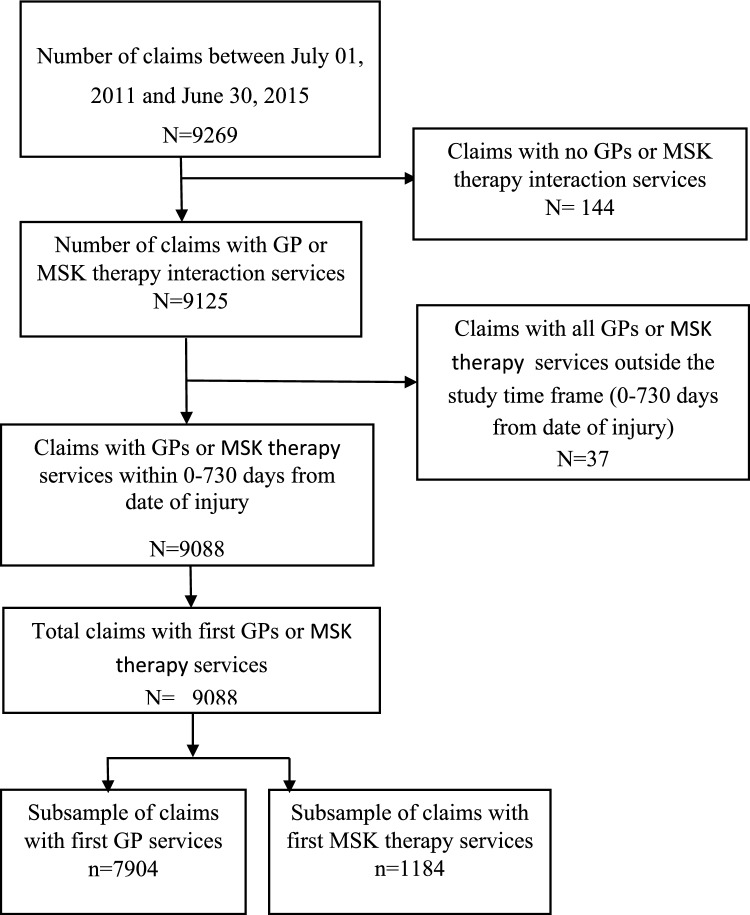
Sample selection procedure

Figure 1 presents the number of LBP claims included from South Australia and Western Australia. 92.0% of claimants received first visit within 30 days of injury dates. Among the included sample, 87.0% of the claimants consulted with GPs as their first healthcare provider, while 13.0% received care from MSK therapists first.

The median time to first visit was 3 (IQR 1–9) days, with GPs being consulted sooner (median 3, IQR 1–8) days than MSK therapists (median 5, IQR 2–14) days (*p* < 0.001). The median time to first visit between South Australia and Western Australia was consistent (median 3 days, IQR 1–8 and 1–10, respectively).

Regarding the median time to first visit among different occupations, the median time to first visit was 4 (IQR 1–10) days for professionals and sales workers each, while the median time to first visit among labourers was 3 (IQR 1–9) days (*p* = 0.002). Workers in remote areas had longer median times to first visit of 4 (IQR 1–12) days, compared to those within major cities, 3 (IQR 1–9) days (*p* = 003) (Supplementary file 1).

### Factors Associated with Time to Initial Healthcare Provider Visit

**Fig. 2:**
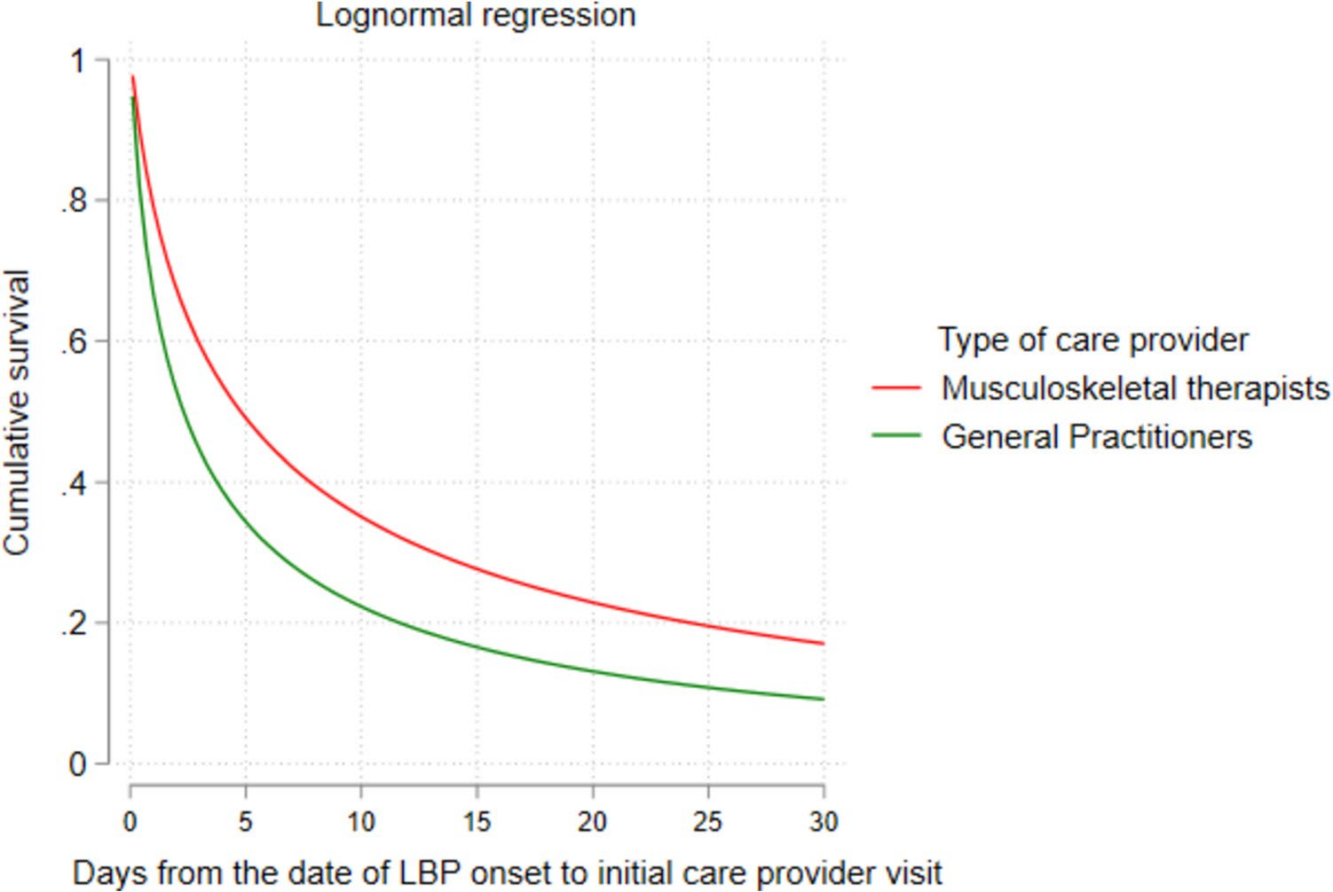
Adjusted survival curve comparing the timing of initial healthcare provider visit by the type of provider since injury date

Table 1 indicates an adjusted lognormal AFT model for the factors influencing the time to first healthcare consultation. In this model, females [TR = 0.87; 95% CI (0.78, 0.97)] compared to males, injuries in 2013 [TR = 0.80; 95% CI (0.72, 0.89)] and 2015 [TR = 0.76; 95% CI (0.65, 0.88)] compared to injuries in 2012, and Machinery Operators and Drivers occupations [TR = 0.87; 95% CI (0.77, 0.99)] compared to labourers experienced a significantly shorter time to first healthcare consultation controlling for all other covariates. Conversely, workers first seeing MSK therapists [TR = 2.12; 95% CI (1.88, 2.40)] compared to GPs, professionals [TR = 1.33; 95% CI (1.09, 1.62)] and sales workers [TR = 1.33; 95% CI (1.09, 1.63)] compared to labourers, and remote workers [TR = 1.27; 95% CI (1.02, 1.60)] compared to workers in major cities were associated with a significantly longer time to first visit. Figure 2 shows the adjusted survival curve comparing time to first visit between GPs and MSK therapists (p < 0.001).

**Table 1:** Factors associated with time to first healthcare provider visit (*N* = 9088)

Variable	Adjusted time ratios (95% CI)	p-value
*Age*		
15–25 years	0.88 (0.77, 1.01)	0.081
26–35 years	0.96 (0.86, 1.07)	0.518
36–45 years	Ref	Ref
46–55 years	1.06 (0.95, 1.19)	0.278
> 55 years	1.09 (0.95, 1.26)	0.18
*Sex*		
Female	0.87 (0.78, 0.97)	0.016
Male	Ref	Ref
*Year of injury*		
2011	0.96 (0.84, 1.09)	0.571
2012	Ref	Ref
2013	0.80 (0.72, 0.89)	< 0.001
2014	0.96 (0.86, 1.08)	0.558
2015	0.76 (0.65, 0.88)	0.001
*Jurisdiction*		
South Australia	0.96 (0.88, 1.05)	0.461
Western Australia	Ref	Ref
*Type first healthcare provider*		
MSK therapists^a^	2.12 (1.88, 2.40)	< 0.001
General practitioners	Ref	Ref
*Occupation*		
Clerical and Administrative Workers	1.00 (0.77, 1.31)	0.955
Community and personal service Workers	1.05 (0.91, 1.20)	0.481
Labourers	Ref	Ref
Machinery operators and drivers	0.87 (0.77, 0.99)	0.036
Managers	1.11 (0.86, 1.44)	0.382
Professionals	1.33 (1.09, 1.62)	0.004
Sales Workers	1.33 (1.09, 1.63)	0.005
Technicians and trades workers	10.99 (0.88, 1.12)	0.967
*Socioeconomic status*		
Most advantaged quintile	1.99 (0.88, 1.11)	0.912
Second to fourth quintiles	Ref	Ref
Most disadvantaged quintile	1.00 (0.88, 1.14)	0.920
*Remoteness*		
Major cities of Australia	Ref	Ref
Regional Australia	1.10 (0.95, 1.27)	0.190
Remote Australia	1.27 (1.02, 1.60)	0.030

^a^MSK therapists include Osteopathy, chiropractor and physiotherapists

*CI* confidence intervals, *N* sample size, *R* reference category

### Factors Associated with Time to First GPs Service

Table 2 presents an adjusted lognormal AFT model with a subsample of workers who saw GPs first. In this model, females [TR = 0.87; 95% CI (0.78, 0.98)] compared to males, individuals with a reported year of injury in 2013 [TR = 0.84; 95% CI (0.75, 0.95)] and 2015 [TR = 0.81; 95% CI (0.69, 0.96)] compared reported to year of injury in 2012, and workers in Machinery Operation and Driving occupations [TR = 0.86; 95% CI (0.75, 0.98)] compared to labourers all had a significantly shorter time to first consultations with GPs. Conversely, after accounting for all other factors, professionals [TR = 1.40; 95% CI (1.13, 1.73)] and sales workers [TR = 1.32; 95% CI (1.06, 1.64)] compared to labourers had a statistically significant longer time to first GPs consultation.

**Table 2:** Associated factors by the type of care provider first seen (*N* = 9088)

Variable	Determinants in those who saw GP first^a^		Determinants in those who saw MSK therapists first^b^	
	Adjusted time ratios (95% CI)	*p*-value	Adjusted time ratios (95% CI)	*p*-value
*Age*				
15–25 years	0.86 (0.74, 1.00)	0.050	1.03 (0.76, 1.39)	0.821
26–35 years	0.94 (0.83, 1.07)	0.390	1.03 (0.81, 1.30)	0.788
36–45 years	Ref	Ref	Ref	Ref
46–55 years	1.08 (0.95, 1.22)	0.192	0.98 (0.77, 1.25)	0.920
> 55 years	1.13 (0.98, 1.32)	0.090	0.84 (0.62, 1.15)	0.301
*Sex*				
Female	0.87 (0.78, 0.98)	0.025	0.89 (0.71, 1.12)	0.331
Male	Ref	Ref	Ref	Ref
*Year of injury*				
2011	0.98 (0.84, 1.13)	0.786	0.98 (0.74, 1.31)	0.930
2012	Ref	Ref	Ref	Ref
2013	0.84 (0.75, 0.95)	0.005	0.62 (0.49, 0.78)	< 0.001
2014	0.99 (0.87, 1.12)	0.957	0.83 (0.66, 1.05)	0.138
2015	0.81 (0.69, 0.96)	0.019	0.53 (0.39, 0.74)	< 0.001
*Jurisdiction*				
South Australia	0.95 (0.86, 1.05)	0.332	1.28 (1.06, 1.54)	0.007
Western Australia	Ref	Ref	Ref	Ref
*Occupation*				
Clerical and administrative workers	1.08 (0.80, 1.44)	0.591	0.69 (0.41, 1.16)	0.168
Community and personal service workers	1.07(0.92, 1.25)	0.342	0.93(0.68, 1.26)	0.663
Labourers	Ref	Ref	Ref	Ref
Machinery operators and drivers	0.86 (0.75, 0.98)	0.026	1.12 (0.86, 1.45)	0.370
Managers	1.09 (0.83, 1.44)	0.500	1.24 (0.73, 2.08)	0.413
Professionals	1.40 (1.13, 1.73)	0.002	0.94 (0.64, 1.38)	0.759
Sales workers	1.32 (1.06, 1.64)	0.011	1.55 (0.95, 2.52)	0.077
Technicians and trades workers	1.03 (0.90, 1.18)	0.599	0.95 (0.73, 1.23)	0.725
*Socioeconomic status*				
Most advantaged quintile	1.04 (0.91, 1.18)	0.513	0.83 (0.65, 1.07)	0.158
Second to fourth quintiles	Ref	Ref	Ref	Ref
Most disadvantaged quintile	0.98 (0.85, 1.13)	0.822	1.3 (0.97, 1.75)	0.073
*Remoteness*				
Major cities of Australia	Ref	Ref	Ref	Ref
Regional Australia	1.10 (0.94, 1.29)	0.220	1.10 (0.81, 1.50)	0.523
Remote Australia	1.25 (0.98, 1.60)	0.065	1.68 (1.04, 2.73)	0.036

^a^7904 (claims who saw GPs first)

^b^1184 (claims who saw MSK therapists first)

*GP* general practitioners, *CI* confidence intervals, *n* sample size, *Ref* reference category

### Factors Associated with Time to First MSK Therapy Services

**Fig. 3:**
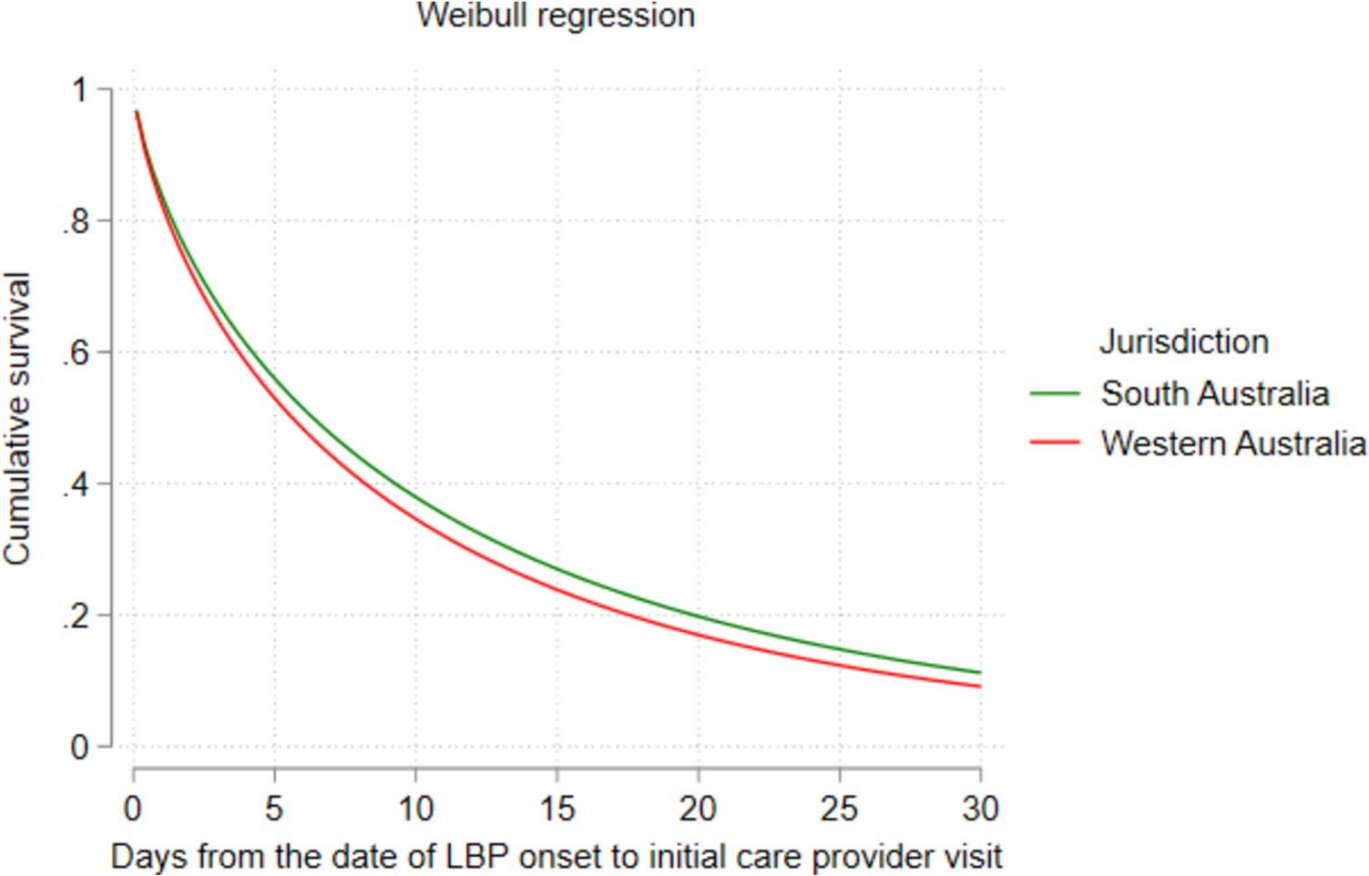
Adjusted survival curve comparing the timing of initial healthcare provider visits in those who consulted MSK therapists first by jurisdiction

An adjusted Weibull AFT model for the subsample of MSK therapy services in those workers who consulted MSK therapists first revealed that the year of injury was associated with a significantly shorter time to first MSK therapy visit. For claims with reported injuries in 2013 and 2015, a significant decrease in time ratio [TR = 0.62; 95% CI (0.49, 0.78)] and [TR = 0.53; 95% CI (0.39, 0.74)], respectively, was observed compared to the 2012 financial year. In this model, jurisdiction emerged as a significant factor associated with the timing of first MSK therapists. The finding demonstrated that a significantly longer initial visit to MSK therapists was observed in South Australia compared to Western Australia [TR = 1.28; 95% CI (1.06, 1.54)]. Figure 3 shows the adjusted survival curve comparing the time to initial visit in those who consulted MSK therapists first by jurisdiction. Furthermore, patients in remote areas experienced a significantly longer time for initial consultation with MSK therapists following their reported low back onset compared to those in major cities [TR = 1.68; 96% CI (1.04, 2.73)] (Table 2).

## Discussion

This study found that a majority of claimants accessed initial visits to healthcare providers within the first 30 days of the reported date of LBP onset, while a small proportion (8%) accessed first workers’ compensation-funded services more than 30 days after injury. The median time from the date of the reported onset of LBP to the first consultation was 3 days. However, this duration was longer in those claimants who consulted MSK therapists first compared to GPs. While no significant inter-jurisdictional variations were observed, several contextual factors were associated with minor differences in time to first visit to a healthcare provider. Claimants from South Australia and those working in remote areas took more time to access their first consultation with MSK therapists. While most injured workers accessed initial healthcare provider visits soon after their injury, there is a need to address barriers to timely access to MSK therapy services and to consider the disparities in certain occupations and geographically disadvantaged individuals.

Although the median difference in time to first service between service types was relatively small (two days), it was statistically significant. Injured workers who saw MSK therapists first compared to those consulting GPs experienced a slightly longer time to initial consultation. Literature reports agree with this finding [24]. This minor difference may hold practical significance when evaluating timing categories independently within the context of MSK therapy (e.g.physical therapists) [40]. A possible explanation could be that most patients are more familiar with choosing to consult GPs funded by compensation insurance initially or may have seen GPs funded by other insurance plans such as Medicare; the Australian national health insurance plan or private insurance. Differences in the scope of practice, such as the authority of healthcare providers to issue initial medical certificates, may also explain the observed discrepancies. For example, only GPs are allowed to write initial certificates of capacity in most Australian states, while MSK therapists are authorised to write subsequent capacity certificates only in certain states, such as Victoria [41]. Further, it is plausible that claimants may lack awareness regarding the potential direct access to consultation with MSK therapists without a referral. Research has shown that direct access by patients to MSK therapists, such as physical therapy is associated with improved patient outcomes and satisfaction and lower utilisation of healthcare resources [42]. Future studies should investigate the effectiveness of the patient-choice-of-care model in workers’ compensation-funded care in facilitating worker outcomes in Australia.

Our analyses identified that individuals in remote locations experienced significantly longer time to healthcare provider visits compared to those in major cities. This finding could be explained by one or a combination of the barriers individuals in remote areas may experience, including a shortage of healthcare professionals or nearby services, distance to healthcare facilities, long waiting times, and difficulties in accessing transportation [43]. Moreover, limited health literacy for individuals in remote areas may further contribute to these disparities [44].

In this study, the consistent trends of significantly shorter time to first care provider visit for females, and for injuries reported in 2013 and 2015, as well as longer time to first care provider visit among professionals and sales workers who consulted GPs first after a reported back pain, could explain the significance of these characteristics not only for the overall time to initial healthcare provider visit but also for the time to first a specific healthcare provider visit. The finding that professionals experienced delayed initial visits is less clear. One possible factor may be due to the differences in access to different healthcare funding options in Australia. Compared to labourers, professionals generally have greater access to private health insurance or may choose to pay out of pocket for initial treatment, especially for minor issues, before deciding to use workers’ compensation schemes. If they don’t formally claim these early services via the workers’ compensation schemes, such services would not appear in the administrative dataset. Another potential factor could be differences in workplace return-to-work policies. Labourers typically work in more physically demanding environments, such as construction, manufacturing, or agriculture, where high-risk tasks—such as heavy lifting, bending, prolonged standing, or repetitive movements are more common. So, workplace policies around early injury reporting and return to work may be stricter in those sectors. Moreover, the pattern of significant difference in time to initial consultation observed by remoteness and year of injury remained consistent in those who consulted MSK therapists first.

Our analysis demonstrated that jurisdiction emerged as a significant factor for those who consulted MSK therapists first. The time to initial consultations with MSK therapists was significantly longer in South Australia compared to Western Australia. Practice variations like differences in implementing return-to-work programmes within workplaces across jurisdictions might contribute to the observed variations in the timing of initial visits to MSK therapists [45–47]. Workplaces with established return-to-work programmes may facilitate earlier access for injured workers [24]. Previous studies have also documented inter-jurisdictional variations in healthcare service use and outcomes for LBP claims [13, 14].

## Limitations and Strength

This study used a large, standardised multi-jurisdiction administrative database from WC authorities in Australia. The study employed robust statistical approaches that estimated the effect of confounders on the probability of accessing medical care timely. However, our study has some limitations. One limitation to consider is that our analyses used data with the reported onset of LBP between 2011 and 2015, and certain policy changes may have occurred since the study period. For example, the South Australian workers’ compensation scheme underwent certain changes in 2015, including eligibility for long-term benefits. However, in both schemes, the legislation related to our primary outcome has not substantially changed. Funding for healthcare related to compensable injury remains a fundamental component of all workers’ compensation schemes in Australia and is available in all jurisdictions for at least the first two years post-claim acceptance. Moreover, thresholds for eligibility for cases of LBP have not changed—eligibility changes have mainly been for other types of injury, such as mental health conditions and some respiratory diseases. So, the policy landscape for healthcare provision in LBP has largely remained unchanged within contemporary workers’ compensation schemes. Future research should examine whether these findings remain consistent in more recent workers’ compensation schemes. Second, the Australian healthcare system provides several healthcare funding options, including ‘Medicare’, ‘out-of-pocket payments’, ‘private health insurance’ or ‘workers’ compensation’ [48]. However, this study examined primarily healthcare funding sources from the WC insurance. While the WC insurance is likely to be the dominant funder during an individual’s claim, it is worth noting that healthcare services around the time of injury, particularly GPs visits, may have been funded by additional sources, most notably Medicare. This could limit our ability to fully understand the actual healthcare use and treatment trajectories of workers’ compensation claimants. Strategies such as data integration, linking and effective information exchange among stakeholders would be beneficial for maintaining comprehensive data. Third, our data do not capture certain clinically important factors, like the severity of symptoms. So, although our analysis considered claims with at least ten consecutive days off work following their LBP onset, we could not account for how this might affect the timing of visits to initial healthcare providers. Finally, this study included working-age individuals receiving workers’ compensation benefits who missed more than two weeks of work following the reported onset of back pain. Therefore, the findings may not reflect the characteristics of the general population or those not receiving workers’ compensation support. The limitations of the multi-jurisdiction administrative database were described elsewhere [27, 28, 32]. Despite these limitations, this study can inform WC insurers and regulators to ensure that all injured workers navigating complex WC systems receive timely, high-quality and equitable WC-funded care regardless of their locations, occupations and the types of first care provider visits (i.e. GPs or MSK therapists).

## Conclusion

The majority of the workers accessed initial care shortly after the onset of reported LBP while a small but significant proportion received later care. The time to initial healthcare consultation following a reported back pain varied significantly by individual, occupational and contextual factors. Personalised care that provides timely and equitable access to services following the onset of compensable LBP can lead to more favourable rehabilitation and return-to-work outcomes for individuals experiencing longer timing of initial healthcare provider visits. Further investigation is required to explore differences in return-to-work outcomes, including work disability duration, following a reported back pain experience.

## Supplementary files

**Supplementary file 1**: First service by service type and population characteristics (N = 9088).

## Supplementary Information

Below is the link to the electronic supplementary material.

Supplementary file1 (DOCX 17 KB)

## Data Availability

The authors cannot distribute the data used in this paper. However, the STATA analysis code used to analyse the data is available upon reasonable request.

## Abbreviations

GPs: General practitioners
LBP: Low back pain
MSK: Musculoskeletal
WC: Workers’ compensation
