# Supplementary material for: Factors Associated with the Timing of Initial Visit to Healthcare Providers for Injured Workers with Low Back Pain Claims: A Multijurisdiction Retrospective Cohort
Source: J Occup Rehabil. 2025 Jan 13;36(1):303–13. doi: 10.1007/s10926-025-10268-5 (PMC12906526; doi:10.1007/s10926-025-10268-5)
Supplement: Supplementary file 1 — Supplementary file1 (DOCX 17 KB) [file 10926_2025_10268_MOESM1_ESM.docx]

**Supplementary table**: First service by service type and population characteristics (N^a^=9088)

| **Characteristics** | | **Type of first service**  **(N %) (row)** | | **Total claims**  **(row)** | **Column % of all claims** | **Median (IQR)b time to first care in days (row)** |
| --- | --- | --- | --- | --- | --- | --- |
|  |  | **General practitioner** | **MSK**  **Therapists*** |  |  |  |
| **All claims** | | 7904 (87.0) | 1184 (13.0) | 9088 | 100 | 3 (1-9) |
| **Gender** | Female | 2598 (86.3) | 412 (13.7) | 3010 | 33.1 | 3 (1-9) |
|  | Male | 5306 (87.3 ) | 772 (12.7) | 6078 | 67.0 | 3(1-9) |
| **Age (years)** | 15-25 | 1046 (88.0) | 143 ( 12.0) | 1189 | 13.1 | 3(1-8) |
|  | 26-35 | 1877 (85.0) | 335(15.0) | 2212 | 24.3 | 3(1-9) |
|  | 36-45 | 2094 (87.3) | 305(12.7) | 2399 | 26.4 | 3(1-9) |
|  | 46-55 | 1892 (87.2) | 277(12.8) | 2169 | 24.0 | 3(1-9) |
|  | 55+ | 995 (89.0) | 124(11.0) | 1119 | 12.3 | 3(1-9) |
| **Year of injury** | 2011 | 1068 (86.7) | 164(13.3) | 1232 | 13.6 | 3(1-9) |
|  | 2012 | 2139 (86.0) | 344(14.0) | 2483 | 27.3 | 3(1-10) |
|  | 2013 | 2228 (88.4) | 292(11.6) | 2520 | 27.7 | 3(1-8) |
|  | 2014 | 1774 (86.2) | 284(13.8) | 2058 | 22.6 | 3(1-9) |
|  | 2015 | 695 (87.4) | 100 (12.6) | 795 | 8.7 | 3(1-8) |
| **Jurisdiction** | WA | 5061 (87.6) | 713 (12.4) | 5774 | 63.5 | 3(1-8) |
|  | SA | 2843 (85.8) | 471(14.2) | 3314 | 36.5 | 3(1-10) |
| **Occupation** | Clerical and Administrative | 200 (85.0) | 36 (15.0) | 236 | 2.6 | 3(1-11) |
|  | Community and Personal Service Workers | 1314 (88.0) | 177 (12.0) | 1491 | 16.4 | 3(1-8) |
|  | Labourers | 1887 (87.8) | 262(12.2) | 2149 | 23.6 | 3(1-9) |
|  | Machinery Operators and Drivers | 1791 (87.4) | 259(12.6) | 2050 | 22.6 | 3(1-8) |
|  | Managers | 225 (86.0) | 37(14.0) | 262 | 3.0 | 3(1-12) |
|  | Professionals | 435 (83.0) | 89(17.0) | 524 | 5.8 | 4(1-10) |
|  | Sales Workers | 408 (89.3) | 49(10.7) | 457 | 5.0 | 4(1-10) |
|  | Technicians and Trade | 1643 (85.6) | 276 (14.4) | 1919 | 21.1 | 3(1-9) |
| **Socio-economic status** | Most advantaged quintile | 1293 (85.4) | 221(14.6) | 1514 | 16.7 | 3(1-8) |
|  | Second to fourth quintiles | 1128 (89.7) | 129(10.3) | 1257 | 13.8 | 3(1-9) |
|  | Most disadvantaged quintiles | 4671 (86.7) | 714(13.3) | 5385 | 59.2 | 3(1-10) |
|  | Missing | 812 (87.0) | 120(13.0) | 932 | 10.3 | 3(1-9) |
| **Remoteness** | Major cities of Australia | 6094 (87.0) | 915(13.0) | 7009 | 77.1 | 3(1-9) |
|  | Regional Australia | 753 (87.8) | 105(12.2)) | 858 | 9.4 | 3(1-9) |
|  | Remote Australia | 251 (85.0) | 44(15.0) | 295 | 3.2 | 4(1-12) |
|  | Missing | 806 (87.0) | 120(13.0) | 926 | 10.2 | 3(1-9) |

Keys: *_=include physiotherapists, chiropractors & osteopaths,_ ^a^_=refers to number of claims,_ ^b^_=interquartile range_
